# Surface Morphology Formation of Edible Holographic Marker on Potato Starch with Gelatin or Agar Thin Coatings

**DOI:** 10.3390/polym12051123

**Published:** 2020-05-14

**Authors:** Aleksandr Podshivalov, Alexandra Toropova, Maria Fokina, Mayya Uspenskaya

**Affiliations:** Bioengineering Research Center, ITMO University, Kronverkskiy Prospekt 49, 197101 Saint Petersburg, Russia; toropa@list.ru (A.T.); mfokina@niuitmo.ru (M.F.); mv_uspenskaya@mail.ru (M.U.)

**Keywords:** hologram, edible coatings, edible films, morphology, potato starch, gelatin, agar

## Abstract

Edible films and coatings based on biopolymers to protect and extend the shelf life of food and medicine can be functionalized, by applying a holographic marker on the coating surface for marking products or sensing storage conditions. In this work, holographic markers were prepared on the surface of thin biopolymer coatings based on starch, gelatin, agar and also starch/gelatin and starch/agar blends by the nanoimprint method from a film-forming solution. The morphology of the surface of holographic markers using optical microscopy in reflection mode was examined, as well as the reasons for its formation using an analysis of the flow curves of film-forming solutions. It was found that the surface morphology of the marker strongly depends on the composition, consistency index of film-forming solution and miscibility of the components. It was shown that the starch/agar film-forming solution at the ratio of 70/30 wt.% has a low consistency index value of 21.38 Pa·s^0.88^, compared to 64.56 Pa·s^0.67^ for pure starch at a drying temperature of 30 °C, and the components are well compatible. Thus, an isotropic morphology of the holographic marker surface was formed and the value of diffraction efficiency of 3% was achieved, compared to 1.5% for the marker made of pure starch. Coatings without holographic markers were analyzed by tensile strength and water contact angle, and their properties are highly dependent on their composition.

## 1. Introduction

Currently, one of the urgent directions in the development of new packaging materials is the creation and study of non-toxic, biocompatible, biodegradable and edible packaging [1]. Such packaging usually consists of biopolymers, blends thereof, as well as biocomposites and can be functionally and active [2]. These materials are used for extending the shelf life of food and drugs, creating forms of drug delivery and partially solving the problem of environmental pollution by non-biodegradable packaging waste [3]. Moreover, such systems may include various edible markers for customer and manufacturer identification, quality control, the monitoring of storage conditions, detection breaking the package integrity and the emergence product spoilage [4].

One of the types of food markers is the edible rainbow holographic marker. Such a holographic marker is a microrelief on the surface of an edible material in the form of a diffraction grating. The period value of such grating is comparable with the wavelengths of the visible spectrum. White light is diffracted on the diffraction grating and produces a bright visual rainbow effect. An edible holographic marker gives the visual multicolor optical effects on the surface of food products without using dyes. This technology can be used for decoration of food products [4,5], the marking of food and pharmaceutical products [6] and for monitoring storage conditions, due to the sensitivity of the markers to humidity and temperature [7,8].

As a rule, the strength of the holographic effect of a marker depends on the period of the diffraction grating *d*, which characterizes the groove density *G*, as well as on the isotropy of the grating microrelief [9]. To evaluate the strength of the holographic effect of a marker, the value of diffraction efficiency *E* is usually used [10].

In the literature, holographic gratings were obtained on the basis of various edible materials and products. Sucrose-based holograms obtained by UV-exposure had a low value of *E_max_* = 0.4%, and their lifetime strongly depended on the relative humidity of the environment [11]. In another work [12], holograms based on sugar and the photosensitive colorant of tartrazine led to *E* = 3.4% and 4.5% for the sugar and sugar/tartrazine blend, respectively. The use of citrus pectin/ferric ammonium citrate photosensitive emulsion as a material for the obtaining of holograms led to a value of *E_max_* = 7.53% [13]. At the same time, sugar-based holograms with the addition of citrus pectin and vanillin as hydrophobic agents have a diffraction efficiency value of *E_max_* = 10% [14]. In one of the authors’ works [15], it was shown that holograms based on fructose/glucose blend with the addition of citrus pectin and vanillin led to a high value of *E_max_* = 44.7%. Holograms based on dichromate gelatin/vanillin photoemulsions [16] were obtained with a wide range of *G* values from 250 to 2000 grooves/mm with an increment of 250 grooves/mm, and showed the highest value *E_max_* = 49%, for a holographic grating with a density of *G* = 500 grooves/mm. UV-exposed holograms from corn honey had a value of *E_max_* = 14% [17]. When the egg albumin/glucose blend was used, the value of *E_max_* = 44% was achieved [18]. In another article [19], embossed holograms were obtained on the surface of corn syrup, with *G* values ranging from 0 to 3000 grooves/mm. It was shown that with increasing *G* values, the diffraction efficiency of holograms decreases linearly.

There are various methods for applying microrelief to the surface of biomaterials. Soft lithography methods include casting a film-forming solution, hot stamping (embossing) [20] and various nanoimprinting methods [21]. The obtaining of holographic diffraction gratings by solution casting on biomaterials, such as plant polysaccharide extracts, different gums, monosaccharides and proteins, was described [22].

The lifetime of edible holograms is a certain period of time, after which the hologram disappears. Considering that edible materials, for example, such as monosaccharides, are very sensitive to environmental humidity [11,12,15,17] and temperature, their lifetime is very different. Some of relaxation processes are appearing in structure of a material during time. For example, the lifetime of hologram made of pineapple juice is only 2 h [23]. The holograms made of shellac can store for more than one year at room conditions, but it disappeared in 15 min at high humidity [7]. The diffraction efficiency of gelatin holograms is reduced by more than 50% over 6 months [24].

One of the relevant materials for creating edible holographic markers is starch from various sources. Due to their greater molecular weight and lower molecular mobility than monosaccharides, starch-based holographic markers will be more susceptible to moisture and temperature. Moreover, a holographic marker on the surface of a biopolymer food coating or drug carrier can be a convenient unified system for quickly monitoring the storage conditions of products. This biopolymer is non-toxic, edible, biodegradable and is derived from renewable plant materials [25]. Chemically, starch is a blend of *α*-glucose polymers, such as linear amylose and branched amylopectin, obtained from agricultural plants. The main advantages of this material are their high solubility in water, the ability to film, a relatively low cost and wide availability in the world market. Starch was mainly used for a long time as a structuring, gelling and water-retaining agent in food products. However, starches are currently relevant materials and have been used to create biodegradable and edible packaging, such as films and foams, disposable dishes, microcapsules for drug delivery systems, as well as for tissue engineering [25,26,27]. Additionally, the adhesive properties of starches and hydrogels based on them were used to create adhesives [28] and conductive adhesives with low distributed electrical resistance [29]. However, starch has a limited application for creating holograms, due to its high fragility and moisture sensitivity due to its polysaccharide structure. 

Mixing starch with water-soluble film-forming biopolymers with a stronger structure and less moisture sensitivity, such as agar or gelatin, will increase its properties. Agar, being a polysaccharide, has a starch-like structure and consists of a blend of galactose polymers, such as agarose and agaropectin, and is extracted from algae. Gelatin is basically a mixture of collagen polypeptides obtained by hydrolysis of animal waste. It was shown that starch-based films, with the addition of gelatins, showed an increase in tensile strength [30,31], elastic modulus [30], water vapor permeability [30,32], solubility in water [33] and oxygen permeability [30]. In turn, when agar was introduced into starch-based films, an increase in the glass transition and melting temperatures [34], tensile strength [34,35,36,37], elastic modulus [34] and water vapor permeability [36] were also observed.

It was shown that there are various techniques for the formation of a holographic microrelief on the surface of various biomaterials. Despite this, to obtain edible highly effective holographic markers, a structured understanding of the processes of microrelief replication with a film-forming solution is necessary. In this regard, the aim of this work was to study the surface morphology formation processes of the edible coatings with a holographic marker based on potato starch, gelatin, agar and starch/gelatin and starch/agar blends on their diffraction efficiency, as well as mechanical strength and wettability.

## 2. Materials and Methods

### 2.1. Materials

Soluble potato starch of puriss./p.a. grade (CAS# 9005-84-9, Ph. Eur.) and a food grade gelatin from hide and bones of bovine and porcine (CAS# 9000-70-8, Ph. Eur.) was obtained from Sigma-Aldrich, Schnelldorf, Germany. Agar Rokogel 2400G produced from red algae (*Rhodophyceae*) was purchased from INDUSTRIAS ROKO, Asturias, Spain. Distilled water was used as a solvent.

### 2.2. Preparation of the Film-Forming Solutions

The initial aqueous solutions of starch, agar and gelatin were prepared at 0.5 wt.% content of dry matter. For this, a biopolymer powder was swelled in distilled water during 10 min at room temperature, followed by gelation and homogenization at 90 °C for the polysaccharide solutions and at 60 °C for the gelatin solution during 10 min. The initial solutions were prepared by constant stirring and maintained at temperature using the C-MAG HS 7 magnetic stirrer, equipped with Ikatron ETS-D6 digital thermometer produced by IKA-Werke, Staufen, Germany. Further, for preparation of the film-forming solutions, the initial solutions are quickly mixed in various ratios of volume fractions starch/gelatin and starch/agar: 100/0, 70/30, 50/50 and 0/100 vol.%, so as to avoid their thermal history. Next, the prepared blends were blended on magnetic stirrer at 70 °C for 5 min.

### 2.3. Dynamic Viscosity Measurements of the Film-Forming Solutions

The viscosity of the solutions was measured using a DV-II + Pro Brookfield digital rotational viscometer manufactured by AMETEK Brookfield, Middleborough, MA, USA equipped with a ULA-EY enhanced adapter and a ULA-49EAY heating jacket for the analyzing of low viscosity liquids. The dynamic viscosity of the biopolymer solutions and their blends were measured in the cooling regime at 70 °C and 30 °C at the range of shear rates from 1 to 250 s^−1^.

### 2.4. Preparation of Thin Coatings with Holographic Markers

**Figure 1:**
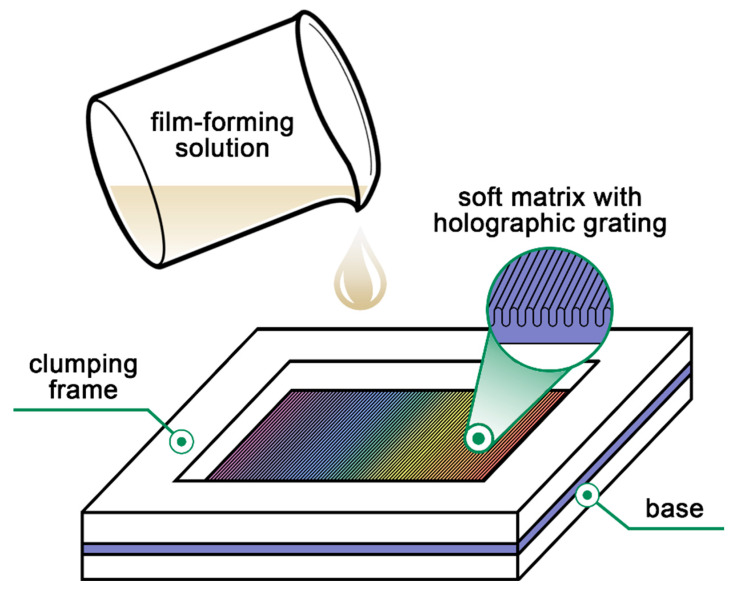
Scheme of the casting mold for the film-forming solution.

To prepare a thin coating with a holographic marker, a nanoimprint technique, such as soft lithography, was used. For this, the special molds were created for casting the film-forming solution. These molds consist of a base with a soft matrix mounted thereon and a clamping frame, which formed a container with dimensions of length x width x height 50 × 50 × 6 mm (Figure 1). The soft matrices were made from a silicone rubber as a submaster replica of a master metal diffraction grating with sinusoidal profile at the groove density of *G* = 600 grooves/mm. For this, the liquid silicone was poured onto the master grating and degassed in a vacuum chamber, followed by solidification under room conditions.

To make thin coatings, the prepared film-forming solutions were casted into the molds and dried at 30 °C during 24 h with continuous air convection, using a UT-4620 laboratory drying oven produced by ULAB, Saint Petersburg, Russia. To ensure a uniform coating thickness, the drying oven was equipped with a bubble level holder. For the tensile and wettability tests, the same coatings without a holographic marker were made, using a silicone template without microrelief.

### 2.5. Optical Microscopy of the Holographic Markers

Morphology of the obtained holographic markers on a coatings surface was analyzed using an STM-6 optical microscope produced by Olympus, Tokyo, Japan in reflection mode at the magnification of ×100. The microscope was equipped with a built-in digital camera and special software.

### 2.6. Diffraction Efficiency Measurements of the Holographic Markers

To estimate the diffraction efficiency of the obtained markers, the intensity of diffracted and transmitted rays of a solid-state single mode diode-pumped laser KLM-635/200 produced by FTI-Optronic, Saint Petersburg, Russia, with a wavelength of *λ* = 635 nm, was measured by TKA-PCM-06 digital photometer produced by TKA Scientific Instruments, Saint Petersburg, Russia in a diffraction order of *m* = −1. Ten intensity measurements were made at different points of the holographic marker for each composition. Further, the diffraction efficiency *E* values are estimated as

(1) $$E=\frac{I_{-1}}{I_{0}}\times 100\%$$

where *I*_−1_ is the beam intensity at *m* = −1; *I*_0_ is the initial beam intensity.

### 2.7. Measurements of Thickness and Tensile Strength of the Coatings

The thickness values of the coatings were measured by digital micrometer with an accuracy of ±0.004 mm produced by TECHRIM, Saint Petersburg, Russia as a mean value of ten different points for each composition. The thickness values are an average of 8 ± 2 μm for all coatings.

The mechanical tensile strength *σ* of the coatings was determined using an electromechanical testing machine Instron 5943, Norwood, MA, USA equipped by 50 N load cell and pneumatic grips for the sample. For these tests, six coating samples of each composition in the form of strips with dimensions of 30 × 10 mm were prepared. Tensile deformation of the samples was carried out at a constant speed of 50 mm/min, which corresponds to ASTM D 882 - 02 standard. The tensile stress during deformation was recorded using Bluehill 3 software.

### 2.8. Measurements of Contact Angle of the Coatings

The surface wettability of the coatings was carried out using a drop shape analyzer DSA 100E produced by KRÜSS, Hamburg, Germany by sessile drop method. During the tests, ten drops of distilled water with an average volume of 2 μL were placed on the surface of the coating samples of each composition. Next, their contours were recorded under room conditions depending on the time in increment of 10 s. The contact angle values *θ* were determined as the mean between the left and right degree values by the ellipse method using the special software Advance.

## 3. Results and Discussion

### 3.1. Rheology of the Film-Forming Solutions

**Figure 2:**
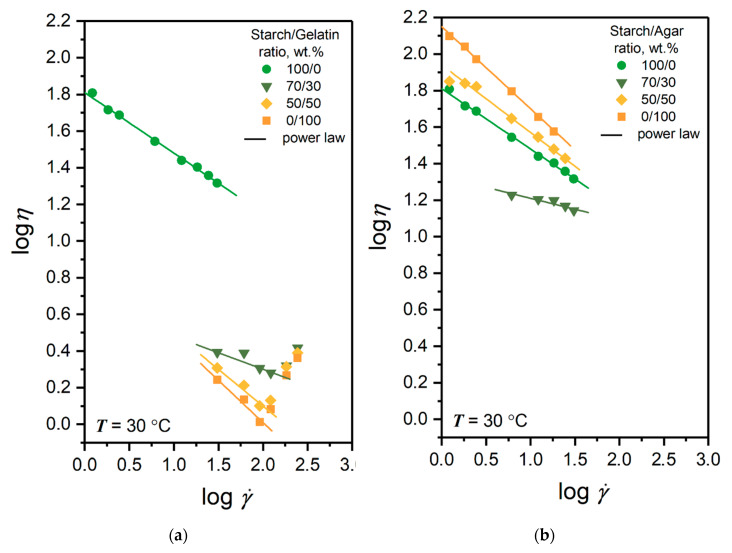
Flow curves of the film-forming solution of (**a**) potato starch, gelatin and their blends and (**b**) potato starch, agar and their blends at a temperature of 30 °C.

To understand the consistency of the film-forming solution during its molding by wet methods, such as casting or spraying of the solution with different shear rates, their dynamic viscosity was measured. Figure 2 shows the flow curves of the potato starch, gelatin, agar, solutions and their blends at a temperature of 30 °C in logarithmic coordinates. Despite the obvious differences in the represented flow curves at the different range of shear rate, which is a drawback of the Brookfield rotational viscometry method for highly diluted solutions, pronounced effects are observed that will be considered.

It can be seen from the figure that effective viscosity of all solutions decreases with an increase in the shear rate, which indicates non-Newtonian behavior. Due to the fact that, in the double logarithmic coordinates the flow curves are close to the linear form, the Ostwald de-Waele power law [38] was used to determine the type of non-Newtonian behavior of the solution effective viscosity *η*

(2) $$\eta =K\cdot {\dot{\gamma }}^{n-1}$$

where *K* is the consistency index, $\dot{\gamma }$ is the shear rate, and *n* is the flow index. The values of *n* and *K* parameters of Equation (2) for the film-forming solutions are presented in Table 1.

The table shows that flow index *n* values for all solutions in the considered ranges of shear rates obey the condition of *n* < 1. Under this condition, in accordance with the theory of the power law, solutions have pseudoplastic behavior and are shear-thinning.

Figure 2a and Table 1 shows that solution of potato starch has a high value of effective viscosity and value of *K* = 64.56 Pa·s^0.67^ at the film-forming temperature, despite a low concentration of 0.5 wt.%. Mixing the starch solution with the gelatin solution at the ratio of 70/30 wt.% leads to a strong decrease in the mean viscosity of the system and its consistency value to *K* = 04.57 Pa·s^0.82^ with respect to the pure starch solution. Mixing the starch and the gelatin solutions in equal proportions (50/50 wt.%) further reduces the average viscosity of the system, making it similar to the pure gelatin solution. However, it is interesting that consistency index value for this solution and for the pure gelatin solution is almost two times higher than value for the solution at ratio of 70/30 wt.%. A similar behavior was observed by other researchers for sago starch/fish gelatin solutions plasticized with glycerol or sorbitol [34], reducing the viscosity of the starch solution from 4 to 3 cP for the blend at a 5:1 ratio. A subsequent increase in the proportion of gelatin also reduced the value of viscosity, but less significantly. In another work [33], the consistency index for the same solutions decreased by more than ten times from a ratio of 1:0 to 3:1.

The observed effect is predictable, and is a consequence of the different molecular mobility of the mixed solutions. The starch solution is sensitive to the temperature used and its molecular mobility at rest is greatly reduced. Its dilution with a small fraction of gelatin solution (30 wt.%) leads to a strong decrease in intermolecular interactions, such as starch–starch and starch–water. As a result, a strong increase in the mobility of the polymer chains at the film-forming temperature is observed. Moreover, such result may indicate that, at this ratio and temperature the solution is structured, and has a phase separation with weak interfacial interaction of the components.

Figure 2b shows the flow curves of the potato starch, agar solutions and their blends. It can be seen that, in general, the effective viscosity and consistency (Table 1) of the solutions with agar is higher than with the gelatin, due to the higher viscosity of the pure agar solution, compared to the pure gelatin solution. The consistency of the pure agar solution is more than double the value of the pure starch solution. However, when mixing the starch and agar solutions in the ratio of 70/30 wt.%, the consistency of the system is significantly reduced relative to individual solutions at *K* = 21.38 Pa·s^0.88^ and the flow index rises to *n* = 0.88. In one of the studies [35], it was shown that at a temperature of 30 °C in a blend (hydroxypropyl cassava/rice starch)/agar in the presence of glycerol, an increase in the agar fraction from 20 to 30 wt.% led to an increase in viscosity from 28 to 33 Pa·s. Such behavior can favorably affect the forming process of the film-forming solution. This result is a consequence of weaker intermolecular interactions of the starch–agar type, compared to starch–starch and agar–agar, which significantly increases the molecular mobility of the system. This is also partially confirmed in studies of the starch/agar blends by the FTIR spectroscopy method of other authors [34,35,39].

### 3.2. Morphology of the Holographic Markers Surface

**Figure 3:**
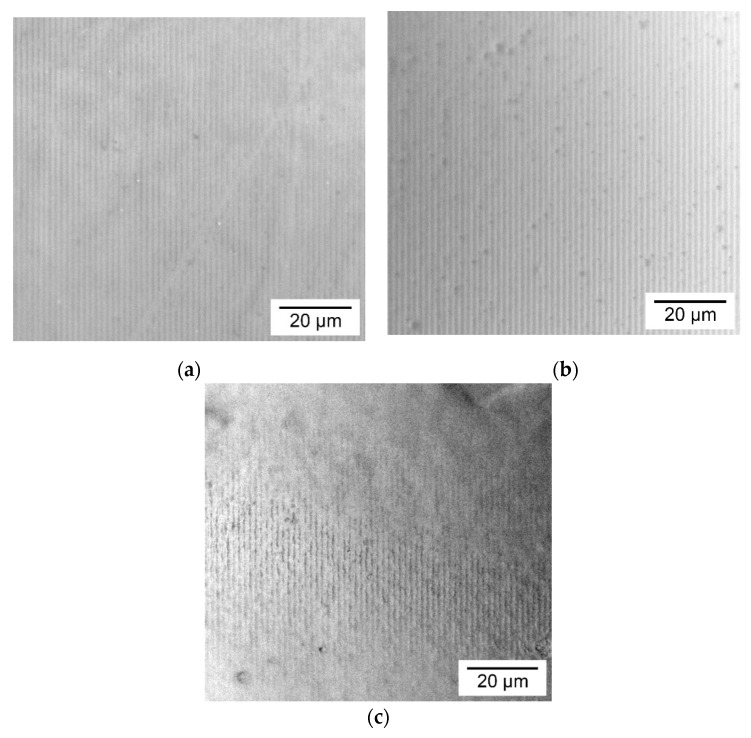
Reflected microphotographs of the holographic markers on the coatings surface made from (**a**) potato starch (**b**) gelatin and (**c**) agar.

The morphology of the holographic markers on the surface of the obtained coatings made from single components is shown in Figure 3.

Images show the dependence of the surface structure of coatings on the type of biopolymer. A similar surface structure of the materials was observed by SEM method for films based on individual starch versus gelatin [30] and starch versus agar [37]. Microphotographs demonstrate the presence of the holographic grating on the coatings surface from all of the considered biopolymers. However, it can be seen that continuity of the dark lines associated with the depth of grating grooves greatly depend on the type of biopolymer. Visual analysis of micrographs shows that deepest grooves has a coating based on gelatin, and then from the potato starch, and worst from the agar. When comparing these observations with the results of the flow curves analysis, it is seen that with an increase in the value of the consistency index, the efficiency of the grating replication from the soft matrix to the coating surface deteriorates. Therefore, a thicker solution is less suitable for the soft matrix replication.

**Figure 4:**
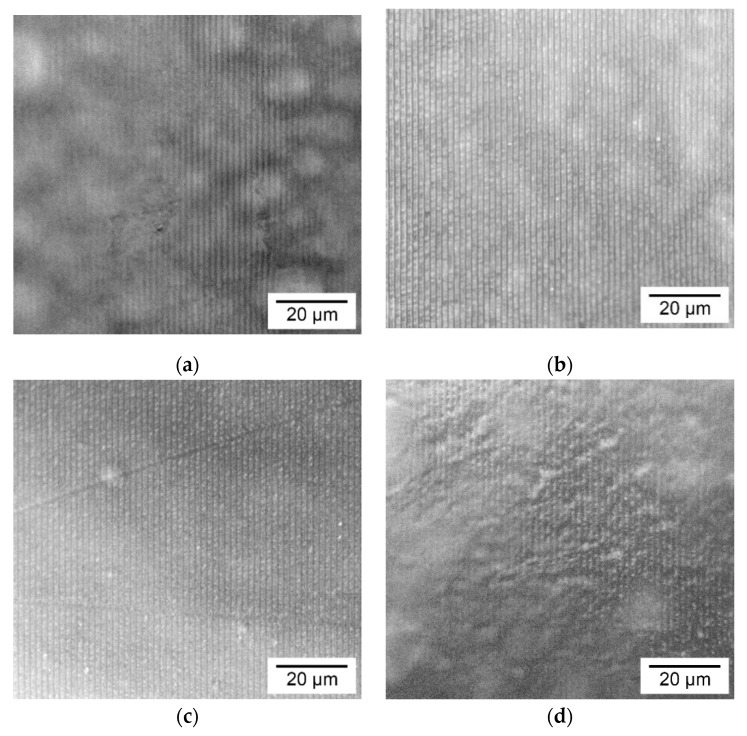
Reflected microphotographs of the holographic markers on the coatings surface made from starch/gelatin blend in the ratios of (**a**) 70/30; (**b**) 50/50 wt.%; and from starch/agar blend in the ratios of (**c**) 70/30; (**d**) 50/50 wt.%.

The morphology of the holographic markers on the coatings surface made from the blends of starch/gelatin and starch/agar solutions at the different ratios is shown in Figure 4. It can be seen that the degree of replication of holographic grating also depends on the consistency of the film-forming solution at the temperature of coating formation. Moreover, a more complicated morphology of the coatings surface is observed.

In Figure 4a, anisotropy of the grating grooves depth is observed, as well as the presence of the light microdomains of a spherical or ellipsoid shape surrounded by dark contours under the coating surface. This structure inside the coating is due to the strong phase separation of the components at the ratio of 70/30 wt.%. The observed effect was also recorded earlier for films based on cassava starch/gelatin (75/25 wt.%) [30] and corn starch/gelatin (80/20 wt.%) [40] blends, using the SEM and synchrotron Fourier transform infrared mico-spectroscopy method, respectively. In our previous study [41], these effects were observed and considered for a reverse system. As shown in Table 1, in this case, the solution has a high value of the flow index *n* = 0.82, which confirms the weaker interaction between the system components and their limited miscibility. As a result, the phase-separated structure of the film-forming solution at the drying temperature leads to a low degree of replication of the soft matrix microrelief.

For the starch/gelatin coating at the ratio of 50/50 wt.% (Figure 4b), there is also a heterogeneous structure inside the coating with the presence of phase separation of the components (combination of dark and light regions), but weaker than the coating at the ratio of 70/30 wt.%. This simplification of morphology is due to the coalescence of aggregates of the starch dispersed phase being suppressed, as confirmed by other SEM studies [30,42]. A similar morphology also was observed for films of starch/gelatin blends in equal proportions [43,44]. As a result, the replication degree of the soft matrix microrelief by this solution is increased.

When being considering the starch/agar blend (Figure 4b,c), the phase separation of components is not observed. The consistency index value of the starch/agar solution at the ratio of 70/30 wt.% is significantly reduced, compared to the solutions of individual components (Table 1). These factors lead to smoothing of the coating surface and high degree of the grating replication (Figure 4c), similar to that based on the pure starch solution (Figure 3a). The same homogeneous morphology was observed for films based on the potato starch/agar [37] and (hydroxypropyl cassava/rice starch)/agar [35] blends at the ratio of 70/30 wt.%, and the components showed high miscibility. However, there are works [34,45] where, at this ratio, a phase-separated morphology is observed. For the starch/agar coating at the ratio of 50/50 wt.% (Figure 4d), the morphology of the coating surface and, accordingly, the holographic grating becomes more complicated, similar to the coating based on pure agar (Figure 3c). This result confirms the viscosity analysis data that, for this ratio of components, the intermolecular interactions of agar–agar type are higher than agar–starch and starch–starch.

### 3.3. Diffraction Efficiency of the Holographic Markers

**Figure 5:**
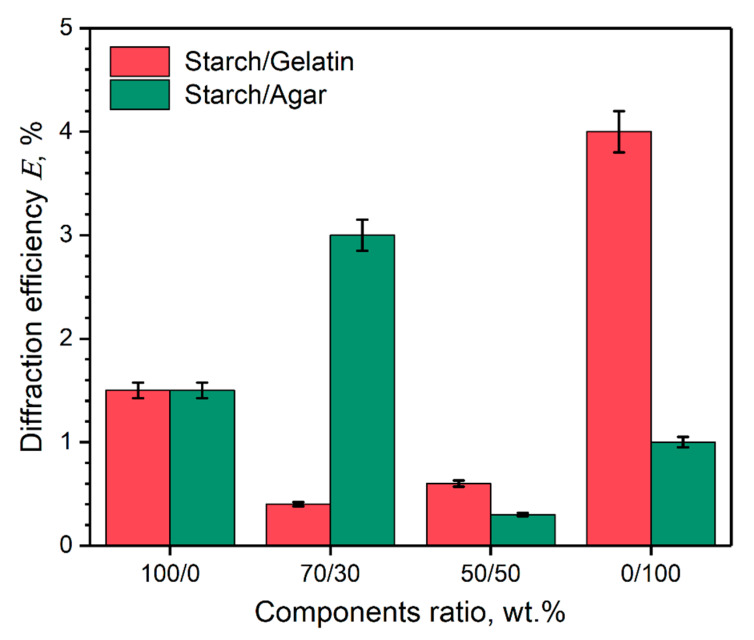
Diffraction efficiency of the holographic markers on the coating surface depends on their composition.

The diffraction efficiency values of the obtained holographic markers on the coatings surface depends on their composition, as shown in Figure 5.

The results of the diffraction efficiency analysis of the holographic markers confirm the results of the visual analysis of their morphology. When comparing the results, it is seen that higher replication degree of the soft matrix microrelief by the film-forming solution, leads to the higher *E* value for the marker. In turn, it was previously considered that degree of grating replication depends not only on the consistency and flow indices of the film-forming solution, but also on its phase structure at the drying temperature. In one study [46], a holographic marker based on albumin with the addition of a photosensitive salt ferric ammonium citrate at a thickness of 15 μm (two times more) had a value of *E* = 2.7%. The markers based on starch/agar blend at the ratio of 70/30 wt.%, as well as from the pure gelatin and starch, which demonstrates that the most isotropic grating grooves and a high degree of replication have the highest *E* values.

### 3.4. Mechanical Tensile Strength of the Coatings

**Figure 6:**
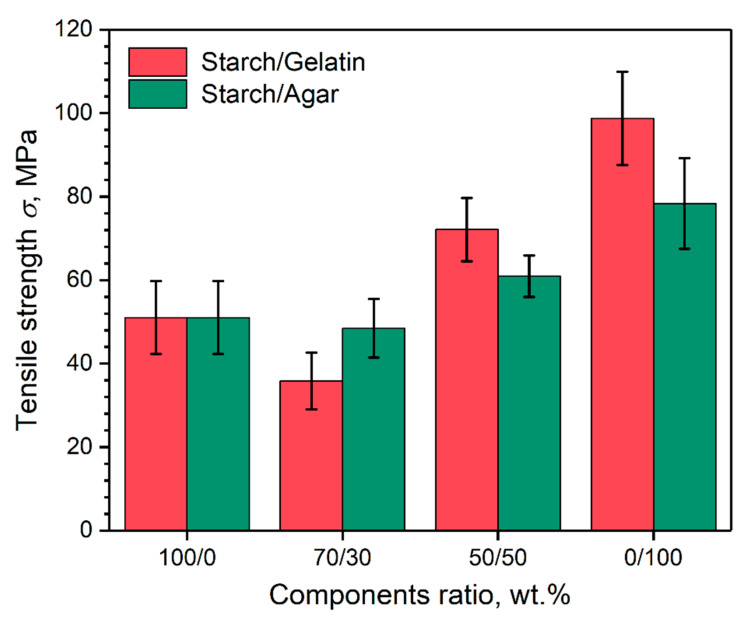
Tensile strength of the coatings depends on their composition.

Figure 6 shows the results of tensile strength analysis of the coatings depends on their composition. 

The graph shows that *σ* values of the coatings based on the potato starch are approximately 1.5–2 times lower than for the coatings based on pure gelatin and agar. The reason for this is the high fragility of the starch coatings, due to the high content of branched amylopectin polymer. When we consider the coatings based on blends of the starch/gelatin and the starch/agar biopolymers, it can be seen that *σ* values depend on the fraction of more durable component. Such behavior of the *σ* values follows from the greater impact of the fraction of stronger component in the coating composition, rather than of the intermolecular or interphase interactions between the components. In other works, a similar behavior of the *σ* value of the starch/gelatin [30,31] and starch/agar [34,35,36] films with the addition of a plasticizer was observed. Despite this, in the case of the starch/gelatin coating at the ratio of 70/30 wt.%, where a strong phase separation of the components was observed (Figure 4a), the lowest value of *σ* = 35.8 MPa was achieved. The phase separation of the components, in this case, leads to a less efficient transfer of the deformation energy to the disparate domains of the phase of the stronger gelatin gel network. Weak phase separation of the components or its absence in coatings based on starch/gelatin and starch/agar blends at the ratio of 50/50 wt.% leads to increased values of *σ* = 72.1 MPa and 60.9 MPa, respectively.

### 3.5. Surface Wettability of the Coatings

**Figure 7:**
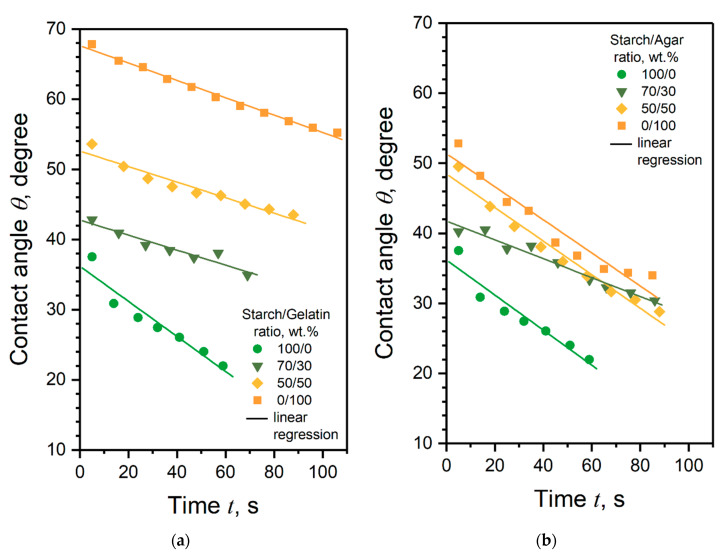
Time dependencies of the water contact angle values of the coatings based on (**a**) potato starch, gelatin and their blends and (**b**) potato starch, agar and their blends.

Figure 7 shows the dependences of the contact angle of the coatings relative to a distilled water, depending on the time of droplet was on the coating surface.

Dependences show that the *θ* value strongly depends on the coating composition. The graph in Figure 7a shows that the wettability of the coatings based on pure gelatin is on average two times lower than coatings based on pure starch. Coatings from the starch/gelatin blend also have greater hydrophobicity than starch coatings. The observed effect is caused by the increased sensitivity of starch to water compared with gelatin, due to the presence of a large number of hydroxyl groups in its structure. Identical behavior of the *θ* values was also observed for the pregelatinized starch/gelatin films, with the addition of camu-camu powder in the presence of sorbitol [31]. Coatings based on starch/agar blends (Figure 7b) have the lower of *θ* values compared to the starch/gelatin coatings, regardless of time. The agar-based coating, like starch, has high wettability, due to its polysaccharide structure. This effect was reported for cassava starch/agar/glycerol films [36].

**Figure 8:**
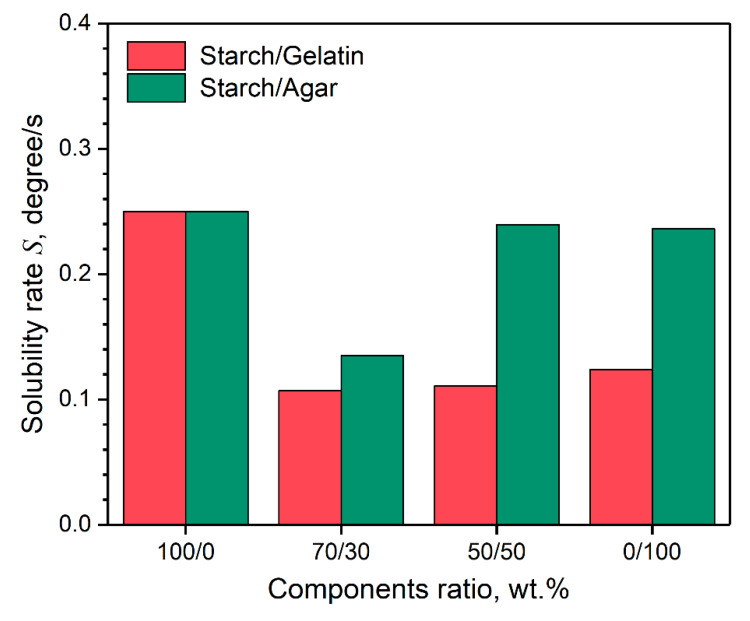
Solubility rate of the coatings depends on their composition.

In the general sense, Figure 7 demonstrates that wettability of the coatings depends not only on their composition, but also on the time of water drop is on their surface. It was found that obtained coatings are not only hydrophilic but also water soluble. As a result, when the drop of water is located on the coating surface, a hole is formed over time. Therefore, the value of solubility rate *S* of the coatings was established using the fitting of the *θ*(*t*) dependencies by the linear regression equation y(x)=a+b⋅x. In this case, the *b* parameter of equation takes the physical meaning of the modulus of solubility rate gradient $b=\left|\frac{\partial \theta }{\partial t}\right|=S$. The value of *S* parameter versus the coating composition are presented in Figure 8.

The graph shows that not only the wettability of the coating surface depends on the composition but also their solubility rate. Coatings containing the gelatin dissolve two times slower than coatings based on polysaccharides and their blends. It was previously shown, for films based on sago starch/fish gelatin in the presence of glycerol, that the solubility of the starch film decreases from 46.56% to 30.19% when mixed with gelatin in a ratio of 3:1 [35]. However, on the image, for the starch/agar-based coating with the component ratio of 70/30 wt.%, solubility has a low rate of *S* = 0.135 degree/s. This may be due to the smoothness of the coating surface (Figure 4c), similar to the coatings with gelatin content. In one of the studies [36], the time dependences of the volume of a drop of water on the surface of cassava starch/agar films with the addition of glycerol were analyzed. The authors showed that for a film with a component ratio of 8:2, a long plateau is observed where the drop volume does not decrease due to the swelling of the material surface. When the ratio of the components in equal shares, this effect was not observed. In another work [35] for a film based on a blend of cassava/rice starch with the introduction of 30 wt.% agar observed a decrease in the rate of solubility of the material by two times. The obtained results are important for the design of coating material, and make it possible to obtain of holographic markers on their surface with the varying degrees of moisture sensitivity.

## 4. Conclusions

In the course of this work, thin biopolymer coatings with holographic markers on the surface were prepared by the soft lithography method. Coatings are prepared from the film-forming aqueous solutions based on potato starch, gelatin, agar, as well as starch/gelatin and starch/agar blends, in various ratios. An analysis of the morphology of the surface of the holographic markers and their diffraction efficiency showed that strength of the holographic effect depends mainly on the coating composition. To determine the effect of coating composition on the strength of holographic effect of the markers, the effective viscosity of the film-forming solutions at the drying temperature was analyzed using the power law model. It was found that the degree of replication of the soft matrix microrelief by film-forming solutions based on starch, agar and their blends deteriorate with increasing value of their consistency index. This leads to a low diffraction efficiency of holographic markers based on them. On the other hand, the degree of replication of the soft matrix for the solutions based on starch/gelatin blends depends more on the strength of the phase separation of the components than on their consistency. The most homogeneous structure within the coating leads to an increase in the diffraction efficiency of such markers. It was shown that mixing potato starch with gelatin or agar in equal proportions increases the mechanical strength of the coatings. Additionally, mixing starch with gelatin leads to a decrease in the wettability and solubility rate of the coatings surface, which is not true for the coatings based on blends of polysaccharides.

The obtained results can be used for stamping of holographic markers with the predicted values of diffraction efficiency, tensile strength and solubility rate on the surface of a continuous packaging biopolymeric coat of a food product or dosage form. Further research will be aimed at studying the influence of temperature and relative humidity on the properties of holographic markers.

## Figures and Tables

**Table 1:** Fitting parameters of the power law model Equation (2).

Components Ratio in Solution, wt.%	*n*	*K*, Pa·s*^n^*
Starch/Gelatin		
100/0	0.67	064.56
70/30	0.82	004.57
50/50	0.60	007.94
0/100	0.54	008.51
Starch/Agar		
100/0	0.67	064.56
70/30	0.88	021.38
50/50	0.63	087.09
0/100	0.55	141.25
